# Effect of *Achyranthes bidentata* Blume on 3T3-L1 Adipogenesis and Rats Fed with a High-Fat Diet

**DOI:** 10.1155/2014/158018

**Published:** 2014-05-14

**Authors:** Sang Deog Oh, Mihyun Kim, Byung-Il Min, Gi Soon Choi, Sun-Kwang Kim, Hyunsu Bae, Chulhun Kang, Deok-Gon Kim, Byoung-Jin Park, Chang Keun Kim

**Affiliations:** ^1^Department of East-West Medicine, Graduate School, Kyung Hee University, Seoul, Republic of Korea; ^2^Department of Medical Science, Graduate School of East-West Medical Science, Kyung Hee University, Yongin, Republic of Korea; ^3^Department of Physiology, College of Medicine, Kyung Hee University, Seoul, Republic of Korea; ^4^Acupuncture & Meridian Science Research Center, Kyung Hee University, Seoul, Republic of Korea; ^5^Division of Homeostatic Development, National Institute for Physiological Sciences, Okazaki, Japan; ^6^Department of Physiology, College of Oriental Medicine, Kyung Hee University, Seoul, Republic of Korea; ^7^Department of Oriental Pediatrics, Kyung Hee Medical Center, Kyung Hee University, Seoul, Republic of Korea; ^8^Department of Family Medicine, CHA University School of Medicine, Seoul, Republic of Korea; ^9^Department of Integrative Medicine, CHA University School of Medicine, 442 Dosan-daero, Gangnam-gu, Seoul 135-948, Republic of Korea

## Abstract

The present study investigated the antiobesity effect of *Achyranthes bidentata* Blume root water extract in a 3T3-L1 adipocyte differentiation model and rats fed with a high-fat diet. To investigate the effect of *Achyranthes bidentata* Blume on adipogenesis *in vitro*, differentiating 3T3-L1 cells in adipocyte-induction media were treated every two days with *Achyranthes bidentata* Blume at various concentrations (1 to 25 **μ**g/mL) for eight days. We found that *Achyranthes bidentata* Blume root inhibited 3T3-L1 adipocyte differentiation without affecting cell viability, and Western blot analysis revealed that phospho-Akt expression was markedly decreased, whereas there was no significant change in perilipin expression. Furthermore, administration of *Achyranthes bidentata* Blume root (0.5 g/kg body weight for six weeks) to rats fed with a high-fat diet significantly reduced body weight gain without affecting food intake, and the level of triglyceride was significantly decreased when compared to those in rats fed with only a high-fat diet. These results suggest that *Achyranthes bidentata* Blume root water extract could have a beneficial effect on inhibition of adipogenesis and controlling body weight in rats fed with a high-fat diet.

## 1. Introduction

Obesity is caused by the accumulation of excessive fat in adipocytes as triglyceride as well as an increased number of adipocytes. Furthermore, this metabolic disease poses high risk for cardiovascular diseases, hypertension, respiratory dysfunction, type 2 diabetes mellitus, certain types of cancer, osteoarthritis, dyslipidemia, ischemic heart disease, and stroke [1, 2].

Differentiation of preadipocytes to mature adipocytes is accompanied by changes in morphology and lipid accumulation within the cells. Therefore, the antiadipogenic effect of potential drug candidates could be represented by the reduction of triglyceride formation in 3T3-L1 adipocyte differentiation. Several signal transduction pathways have been found to play an important role in adipocyte differentiation and lipolysis. PKB/Akt is a downstream signal of phosphatidylinositol-3-kinases (PI-3-Kinases) pathway that is essential for adipogenesis. For example, inhibition of PI-3 kinase by an inhibitor blocked insulin-induced 3T3-L1 adipocyte differentiation [3, 4]. On the other hand, protein kinase A exerts its lipolysis effect through its downstream target, perilipin, which is highly expressed during adipogenesis. Moreover, it is well known that overexpression of perilipin A decreases lipolysis by hormone-sensitive lipase (HSL) and lipid turnover resulting in increases in storage of triglyceride in adipocytes [5, 6].

Despite the fact that medication for the treatment of obesity has short-term benefits for weight loss by suppression of appetite, they are often associated with side effects and have potential for drug abuse. Rebound weight gain is also considered a major side effect after the cessation of medication use [7]. Recently, many herbal medicines and their derivatives have drawn attention because of their beneficial effect on the treatment of obesity without causing significant side effects or low risk for addiction [8]. For instance, a number of animal studies showed that many herbal medicines such as ginseng [9],* Rhus verniciflua *Stokes [10],* Juniperus chinensis *[11], and* Momordica charantia *[12] are effective in reducing weight gain in high fat-induced obesity animal models.

*Achyranthes bidentata *Blume (ABB), a member of Amaranthaceae, has been traditionally used for fever, asthma, rheumatism, headache, and hypertension [13]. Recently, it has been reported that ABB extract prevents glutamate-induced cell damage in cultured hippocampal neurons and induces nerve growth and neural differentiation [14–16]. However, till now, effects of ABB on adipogenesis and its molecular mechanism have not been studied. The present study was designed to investigate the potential antiadipogenic effects of ABB water extract in 3T3-L1 adipocyte differentiation. In addition to these cell studies, ABB was orally administered to high-fat-diet-induced obese rats to demonstrate its beneficial effect on obesity* in vivo*.

## 2. Materials and Methods

### 2.1. Materials

Ten herbs including* Achyranthes bidentata *Blume (ABB), were purchased from the Oriental Medicine Department of Kyung Hee Medical Center, Kyung Hee University (Seoul, Korea). Dulbecco's modified Eagle's medium (DMEM) and antibiotics were obtained from Gibco/BRL (Grand Island, NY, USA). Oil red O, 3-(4,5-dimethylthiazol-2-yl)-2,5-diphenyltetrazolium bromide (MTT), isobutylmethylxanthine (IBMX), dexamethasone, and insulin were purchased from Sigma (St. Louis, MO, USA). All antibodies were obtained from Santa Cruz Biotechnology (Santa Cruz, CA, USA) and Abcam (Cambridge, UK).

### 2.2. Preparation of Herbal Extracts

The dried herbs were soaked in cold distilled water overnight and then extracted by boiling for 3 hours at 100°C. The aqueous extracts were filtered through distilled gauze and concentrated by an evaporator. The extracted herbal medicines were dried with a freeze dryer and stored at −20°C until the experiment was performed.

### 2.3. 3T3-L1 Cell Culture and Adipocyte Differentiation

Murine 3T3-L1 preadipocytes (ATCC, Manassas, VA, USA) were maintained in DMEM supplemented with 10% (v/v) BCS and 1% (v/v) antibiotics in a humidified atmosphere of 95% air and 5% CO_2_ at 37°C. The cells were seeded at a density of 2.4 × 10^5^ cells/cm^2^ and grown until cells reached confluency. Differentiation to adipocytes was induced by adipocyte-induction media I (DMEM containing 10% [v/v] FBS, 1% [v/v] antibiotics, 0.5 mM IBMX, 1 *μ*M dexamethasone, and 10 *μ*g/mL insulin). After three days, cells were changed to adipocyte-induction media II (DMEM containing 10% [v/v] FBS, 1% [v/v] antibiotics, and 10 *μ*g/mL insulin) for 5 additional days. All media were changed every 2 days.

### 2.4. Treatment of Herb Extracts on 3T3-L1 Cells

Herb extracts were dissolved in adipocyte-induction media and filtered through 0.2 *μ*m-pore syringe filters. Differentiating 3T3-L1 cells were treated every 2 days with a herb in adipocyte-induction media at various concentrations (1 to 25 *μ*g/mL) for 8 days. The concentration used in the experiment was based on the dry weight of the extract.

### 2.5. Oil Red O Staining

3T3-L1 adipocytes that were treated as described above were washed with phosphate-buffered saline (PBS, pH 7.4) and fixed with 4% paraformaldehyde (PFA). After incubation for 1 hour, cells were rinsed with PBS and were stained with oil red O solution (0.2% [w/v] in isopropanol) for 1.5 hours at room temperature. To obtain the image of cell lipid droplets stained with oil red O, appearance was recorded by microscopic at 200x magnification with an Axiovert S 100 (Carl Zeiss) equipped with an AxioCam. Subsequently, oil red O dye in lipid droplets was eluted into isopropanol and the absorbance was measured at 490 nm using an ELISA reader (Emax, USA) (*n* = 4).

### 2.6. Cell Viability Assay

Cell viability was determined by MTT assay. On day 8, 100 *μ*L of MTT solution (5 mg/mL) was added to differentiated 3T3-L1 cells. After incubation at 37°C for 2 hours, the synthesized formazan crystals were dissolved in dimethyl sulfoxide (DMSO). Finally, absorbance was measured at 570 nm using an ELISA reader (Emax, USA) (*n* = 4). Data was calculated as a percentage of MTT compare to control cells.

### 2.7. Western Blot Analysis

On day 8 after the initiation of differentiation treated with various concentrations of ABB (0–25 *μ*g/mL), cells were rinsed three times with cold PBS and scraped into lysis buffer (20 mM Tris-HCl [pH 7.4], 150 mM NaCl, 10% glycerol, 2% Nonidet P-40, 1 mM EDTA, 20 mM sodium fluoride, 30 mM sodium pyrophosphate, 0.2% sodium dodecyl sulfate (SDS), 0.5% sodium deoxycholate, 1 mM phenylmethylsulfonyl fluoride (PMSF), 1 mM dithiothreitol (DTT), and 1 mM sodium vanadate). The cell lysate was centrifuged for 20 min at 12,000 ×g and the supernatant was collected. Each protein extract (5, 10, or 20 *μ*g) was loaded on 10% SDS-polyacrylamide gel and the separated proteins were blotted onto 0.2 *μ*m polyvinylidene difluoride (PVDF) membranes (Bio-Rad, Hercules, CA, USA) using a semidry transfer apparatus (Trans-Blot SD; Bio-Rad). The membranes were then incubated with primary antibody (Akt-p [1 : 1000], Akt [1 : 2000], perilipin [1 : 2000], and actin [1 : 1000]) diluted in blocking solution at 4°C overnight. Membranes were probed with horseradish peroxidase-labeled secondary antibody (anti-goat IgG or anti-rabbit IgG) for 1 hour at room temperature. After several washing steps, the immunoreactive bands were detected by chemiluminescence using West-one (iNtRON Biotechnology).

### 2.8. Image Analysis

All image analysis was performed with ImageMaster 2D Elite software, version 3.1 (Amersham Pharmacia Biotechnology). The signal intensity of each band was analyzed by lowest background subtraction and normalized by band intensity of Akt (for Akt-p) and actin (for perilipin).

### 2.9. *In Vivo *Antiobesity Activity

Male Sprague-Dawley rats aged 6-week-old, weighing 170~190 g (Samtaco, Seoul, Korea), were housed four per cage in a laminar airflow room maintained at a temperature of 22 ± 1°C and a relative humidity of 55 ± 1%. After the animals were given a standard laboratory diet for one week, they were randomly divided into a normal diet group (ND) (*n* = 6), high-fat diet group (HFD) (*n* = 6), and a high-fat diet + ABB group (HFD + ABB) (*n* = 6).  Table 1 shows the HFD composition. Rats in the HFD group were orally administered saline once a day, while rats in the HFD + ABB group were orally administered 0.5 g ABB/kg body weight in saline. All experimental rats were allowed free access to the diet and water during the experimental period. The research was conducted in accordance with the internationally accepted principles for laboratory animal use and care as found in US guidelines (NIH publication #85-23, revised in 1985). Food intake and body weights were recorded daily for 6 weeks. The rats were euthanized by anesthetic overdose and blood was taken for triglyceride, total cholesterol, LDL cholesterol, and HDL cholesterol analysis (ARC Laboratory, South Korea).

### 2.10. Statistical Analysis

Data are presented as mean ± SEM. Analysis was performed by ANOVA, with Bonferroni's test for multiple comparisons, and by *t-*test, using SPSS 11.0 software. A *P* value of <0.05 was considered statistically significant.

## 3. Results

### 3.1. Effects of 10 Herbal Medicines on Lipid Accumulation

**Figure 1:**
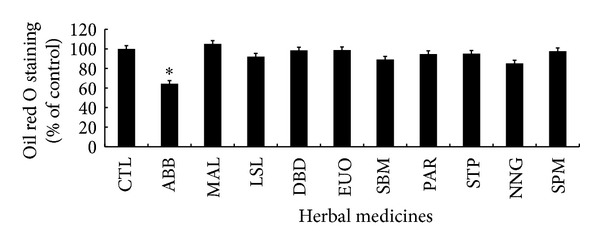
Effect of various herbal medicines on cellular lipid droplets in cultured 3T3-L1 adipocytes. Differentiating 3T3-L1 cells were treated every 2 days with 10 *μ*g/mL of herbal medicines in adipocyte-induction media and stained with oil red O on day 8. The accumulation of lipid contents was detected at 490 nm after oil red O dye was dissolved in isopropanol. Results represent the mean ± SEM of four independent experiments (*P < 0.005 versus control). CTL (control),* Achyranthes bidentata *Blume (ABB),* Morus alba *Linn (MAL),* Leonurus sibiricus *Linn (LSL),* Dioscorea batatas *Decaisne (DBD),* Eucommia ulmoides *Oliver (EUO),* Scrophularia buergeriana *Miquel (SBM),* Phellodendron amurense *Ruprecht (PAR),* Nelumbo nucifera *Gaertner (NNG),* Schizonepeta tenuifolia *Briquet (STP),* Siegesbeckia pubescens *Makino (SPM).

The antiadipogenic effects of 10 herbal medicines were determined by oil red O staining. Postconfluent 3T3-L1 preadipocytes were treated with 10 *μ*g/mL of a herbal medicine every 2 days, while the preadipocytes were differentiated into adipocytes.  Figure 1 shows that, in contrast to control cells and other herb-treated cells, the cells treated with* Achyranthes bidentata *Blume (ABB) demonstrated a significant reduction of lipid accumulation. The oil red O staining result of ABB-treated cells showed a 36% inhibitory effect on lipid content in 3T3-L1 adipocytes. Therefore, ABB was subjected to further analysis to investigate its possible antiadipogenic effect on adipocyte differentiation.

### 3.2. Effect of* Achyranthes bidentata* Blume on Cell Viability

**Figure 2:**
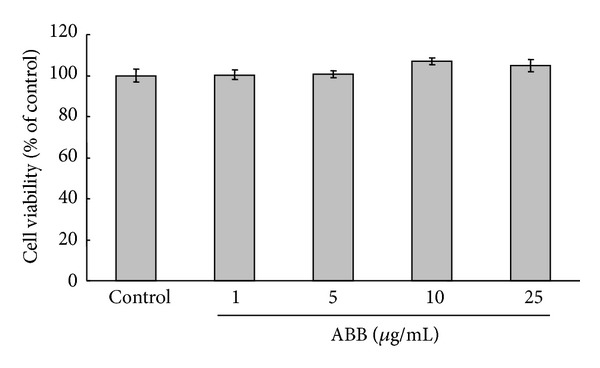
Effect of ABB on cell viability in cultured 3T3-L1 adipocytes. Differentiating 3T3-L1 cells were treated every 2 days with 0–25 *μ*g/mL of ABB for 8 days in adipocyte-induction media. Cell viability was calculated as a percentage of MTT metabolisms in controls. Results represent the mean ± SEM of four independent experiments.

To examine the cell viability of ABB on 3T3-L1 adipocyte differentiation, postconfluent 3T3-L1 preadipocytes were maintained in adipocyte-induction media and were exposed to various concentrations of ABB (1, 5, 10, and 25 *μ*g/mL) and they were treated every 2 days with 0–25 *μ*g/mL of ABB for 8 days in adipocyte-induction media. As shown in Figure 2, none of the concentrations of ABB affected cell viability in 3T3-L1 adipocytes, showing that ABB has no cytotoxic effects at concentrations up to 25 *μ*g/mL in 3T3-L1 adipocytes.

### 3.3. Effect of* Achyranthes bidentata* Blume on Lipid Accumulation

**Figure 3:**
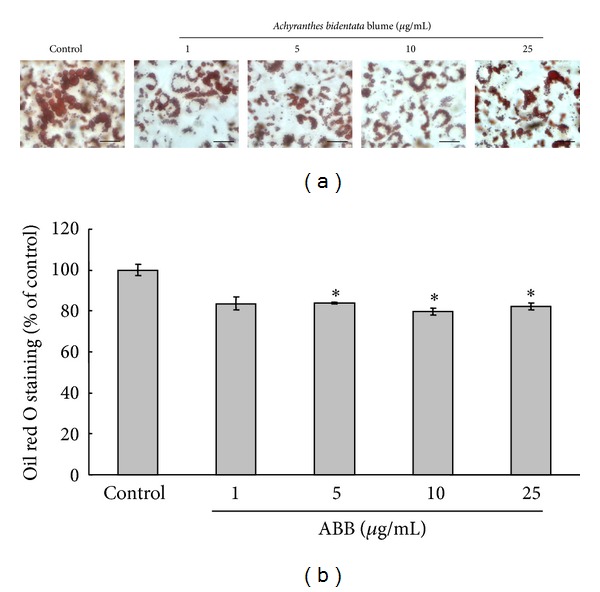
Effect of ABB on cellular lipid droplets and cell viability in cultured 3T3-L1 adipocytes. Differentiating 3T3-L1 cells were treated every 2 days with 0–25 *μ*g/mL of ABB for 8 days in adipocyte-induction media. (a) Intracellular lipids were stained with oil red O (scale bar; 20 *μ*m). (b) The accumulation of lipid contents was detected at 490 nm after oil red O dye was dissolved in isopropanol. Results represent mean ± SEM of four independent experiments (*P < 0.05).

The effects of ABB on adipocyte differentiation and cellular lipid drops are shown in Figure 3. On the 8th day of treatment with various concentrations of ABB (1, 5, 10, and 25 *μ*g/mL) in adipocyte-induction media, intracellular lipid drops were examined by oil red O staining. Control cells showed a large amount of lipid drop accumulation, but the cell treated with 1 *μ*g/mL, 5 *μ*g/mL, 10 *μ*g/mL, and 25 *μ*g/mL of ABB showed relatively smaller amounts of lipid droplet accumulation as observed by reduced oil red staining (Figure 3). The reduction of lipid accumulation is a result of an inhibitory effect of ABB on adipocyte differentiation in 3T3-L1. The cell viability result showed that ABB had no cellular toxicity (Figure 2).

### 3.4. Effects of* Achyranthes bidentata* Blume on Expression of Phospho-Akt and Perilipin

**Figure 4:**
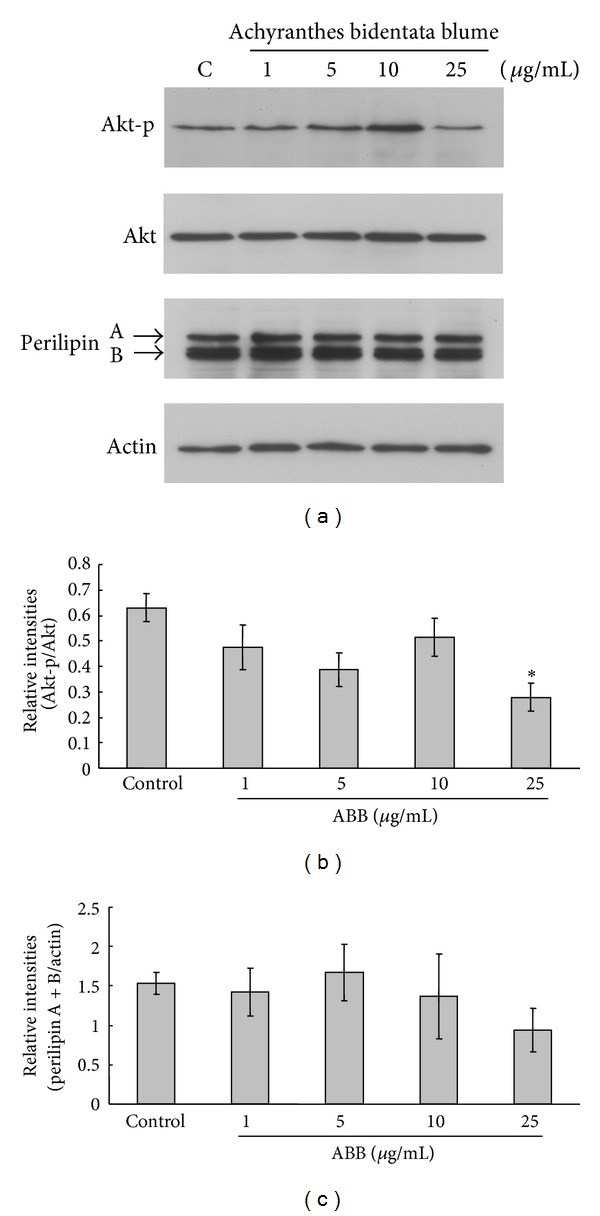
Effects of ABB on Akt-p and perilipin A/B expression. Differentiating 3T3-L1 cells were treated every 2 days with 0–25 *μ*g/mL of ABB for 8 days in adipocyte-induction media. (a) Expression level of Akt-p and perilipin was normalized by Akt and actin and quantified as relative intensities (b) and (c). Results represent mean ± SEM of three independent experiments (*P < 0.05).

The results of oil red O and cell viability showed that ABB inhibits intracellular lipid accumulation without affecting cell viability in 3T3-L1 adipocytes. Next the effect of ABB on the expression of adipogenic and lipolysis factor in 3T3-L1 adipocytes was examined. To examine the molecular mechanisms and whether inhibition of lipid accumulation in adipocyte differentiation resulted from ABB-mediated expression of adipogenic protein, the effect of ABB on the level of phosphorylated Akt (phospho-Akt) was first examined by Western blot from 3T3-L1 cell lysates treated with ABB at various concentrations (1, 5, 10, and 25 *μ*g/mL). Serine/threonine kinase Akt is a downstream target of phosphatidylinositol-3-kinase (PI3-Kinases) in adipocyte differentiation [3, 4]. As shown in Figure 4, treatment with 25 *μ*g/mL of ABB decreased the level of phospho-Akt in comparison to control cells. Since intracellular lipid accumulation in the differentiation of 3T3-L1 preadipocytes into adipocytes can also decrease by the acceleration of lipolysis along with decreased expression of the lipid droplet-associated protein perilipin [5, 6], the next examination was whether ABB alters perilipin expression in 3T3-L1 adipocytes. As shown in Figure 4, Western-blot result from 3T3-L1 cell lysates treated with various concentrations (1, 5, 10, and 25 *μ*g/mL) of ABB in 3T3-L1 adipocytes showed that ABB had no significant effect on perilipin protein expression. Taken together, these results suggest that ABB effects a reduction of lipid accumulation through modulation of Akt signaling pathway in 3T3 -L1 adipocytes, and ABB has no effect on the alteration of lipolysis* via* perilipin expression.

### 3.5. Effect of* Achyranthes bidentata* Blume on Rats Fed with High-Fat Diet

**Figure 5:**
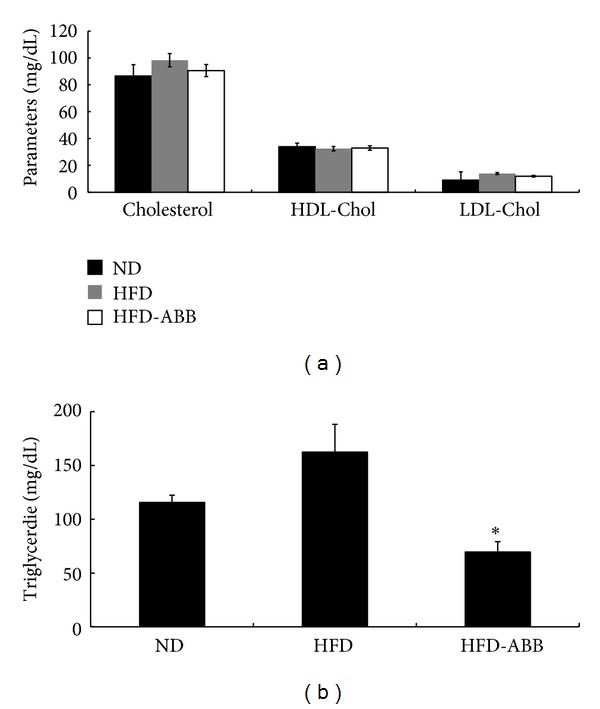
Effect of ABB on lipid profiles. (a) Effect of ABB on total cholesterol, HDL-Chol, and LDL-Chol and (b) triglyceride level in normal diet (ND), high fat diet (HFD), and HFD + ABB-fed rats groups after six weeks. Results represent mean ± SEM (*P < 0.05 versus HFD, *n* = 6 per group).

**Figure 6:**
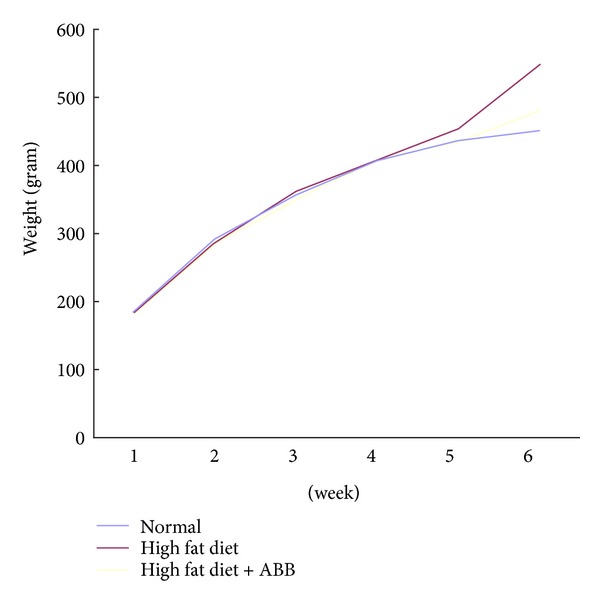
Growth curve. Weight gain of rat in normal diet group (ND) (*n* = 6), high-fat diet group (HFD) (*n* = 6), and a high-fat diet + ABB group (HFD + ABB) (*n* = 6).

To investigate the antiadipogenic effect of ABB* in vivo*, ABB (0.5 g/kg per day) was orally administrated to HFD-induced rats for 6 weeks (Figure 6).  Table 2 shows that there was no significant difference in food intake between HFD and HFD + ABB groups. However, the final body weight was significantly lower in the HFD + ABB group when compared to those of the HFD group. Moreover, the concentration of triglyceride in plasma was remarkably decreased by ABB administration compared to rats fed only the HFD (Figure 5(b)). Although there was no significant difference observed in total cholesterol, HDL, and LDL (Figure 5(a)), oral administration of ABB suppressed HFD-induced weight gains as well as triglyceride concentrations in plasma without affecting food intake.

## 4. Discussion

Obesity has become a major health problem worldwide. It is associated with heart diseases, hypertension, cancer, and type 2 diabetes [17]. Even though alternative therapies for obesity such as herbal medicine treatments have long been used in eastern cultures, few herbal medicines have been investigated to prove their efficacy and mechanisms compared to western drugs.

The present study demonstrated for the first time that* Achyranthes bidentata *Blume (ABB) root water extract causes a significant inhibition of lipid accumulation without cytotoxicity (Figure 2) and a decrease in the expression of Akt phosphorylation in 3T3-L1 adipocytes (Figure 3), suggesting that ABB extract may block adipogenesis by inhibition of the Akt pathway, which is a major signal pathway for adipocyte differentiation activated by insulin/IGF signaling. Since ABB inhibited lipid accumulation during differentiation of 3T3-L1 preadipocytes into adipocytes, high-fat-diet (HFD)-induced obese rats were orally administrated ABB water extract for 6 weeks to verify the antiobesity effect of ABB* in vivo*. The animal study revealed that the body weight increase caused by a HFD was significantly decreased by ABB, but ABB did not change food intake (Table 2). One possible explanation would be that ABB may impair adipogenesis and decrease lipid accumulation in adipocytes. Although examinations were not made of phosphorylated-Akt expression in adipocytes from ABB-administrated rats, it is supported by the* in vitro* study that showed that ABB causes a decrease in lipid accumulation by inhibition of Akt phosphorylation in 3T3-L1 adipocytes. It is also possible that ABB may induce increased energy expenditure in HFD-induced obese rats. However, this must be confirmed by further investigation.

ABB is known to contain a variety of bioactive components including steroids, alkaloids, saponins, flavonoids, and phenols [18, 19]. It is well known that phenolic acids and flavonoids have pharmacological activities such as antioxidant, anti-inflammatory, and anticancer activities [20, 21]. Recently, Hsu and Yen [22] investigated the effect of phenolic acids and flavonoids on the inhibition of adipogenesis in 3T3-L1 adipocytes. Interestingly, among 15 phenolic acids and 6 flavonoids tested in that study,* o*-coumaric acid and rutin showed the highest inhibition of GAPDH activity and intracellular triglyceride accumulation in 3T3-L1 adipocytes. However, further studies should be performed to identify what component(s) in ABB and what changes in another adipogenesis marker(s) is (are) responsible for its antiadipogenesis in this study.

The results obtained in the present study showed antiadipogenic effects of ABB on preadipocytes into adipocyte differentiation and molecular mechanisms in 3T3-L1 cells, which was confirmed in the HFD-induced obese rat model. ABB water extract inhibited lipid accumulation in differentiating 3T3-L1 adipocyte by a decrease in the protein expression of phosphorylated Akt. Moreover, body weight gain and plasma triglyceride levels were effectively decreased in HFD-fed rats with ABB (g/kg) administrated for 6 weeks. Taken together, these results suggest that ABB could have a beneficial effect by inhibition in adipogenesis and reducing body weight gain in rats fed a HFD.

## Figures and Tables

**Table 1:** Composition of experimental diets.

% Content	Normal diet	High-fat diet
Casein-80-mesh	19.0	25.8
L-cystine	0.3	0.4
Corn starch	29.9	0.0
Maltodextrin 10	3.3	16.2
Sucrose	33.2	8.9
Cellulose, BW200	4.7	6.5
Soybean oil	2.4	3.2
Lard	1.9	31.7
Mineral mix S10026	0.9	1.3
Dicalcium phosphate	1.2	1.7
Calcium carbonate	0.5	0.7
Potassium citrate	1.6	2.1
Vitamin mix V10001	0.9	1.3
Choline bitartrate	0.2	0.3

**Table 2:** Weight gain and food intake in normal diet, high-fat diet, and high-fat diet + ABB groups for six weeks.

Ingredient (%)	Normal diet	High-fat diet	High-fat diet + ABB
Body weight (g)			
Initial	183.9 ± 2.0	184.5 ± 2.8	183.8 ± 1.8
Final	449.5 ± 13.3	549.2 ± 28.6	480.9 ± 15.1*
Food intake (g)	183.0 ± 2.9	145.7 ± 1.8	146.9 ± 2.3

Data represents the mean ± SEM, ABB: *Achyranthes bidentata *Blume (0.5 g/kg body weight/daily). *P < 0.05, when compared to the high-fat diet group (*n* = 6 per group).
